# Decompressive Laminectomy for Neurological Complications of Spondylodiscitis: A Case Series of 15 Patients

**DOI:** 10.3390/jpm16090440

**Published:** 2026-08-22

**Authors:** Andrea Bruno, Antonio Meola, Stefano Di Bella, Leonello Tacconi

**Affiliations:** 1 Neurosurgery Unit, Department of Medicine, Surgery and Health Sciences, University Hospital and Health Services of Trieste—ASUGI, University of Trieste, Strada di Fiume, 447, 34149 Trieste, Italy; andrea.bruno@asugi.sanita.fvg.it (A.B.); leonello.tacconi@asugi.sanita.fvg.it (L.T.); 2 Infectious Diseases Unit, Department of Medicine, Surgery and Health Sciences, University Hospital and Health Services of Trieste—ASUGI, University of Trieste, Via Gatteri 25/1, 34125 Trieste, Italy; stefano.dibella@asugi.sanita.fvg.it

**Keywords:** spondylodiscitis, vertebral osteomyelitis, decompressive laminectomy, epidural abscess, spinal infection, neurological outcome, personalized medicine

## Abstract

**Background/Objectives**: Surgical indications for spondylodiscitis are controversial, particularly when epidural extension and evolving neurological compromise are present. This study aimed to describe the clinical, neurological, pain-related, and inflammatory outcomes of patients with spondylodiscitis treated with decompressive laminectomy without spinal stabilization, at a single tertiary center. **Methods**: We retrospectively reviewed 15 consecutive patients with spondylodiscitis who underwent decompressive laminectomy at the University Hospital of Trieste between January 2017 and December 2023. Inclusion required at least 6 months of clinical and laboratory follow-up. Patients treated with acute vertebral stabilization were excluded. Collected data included spinal level, timing from diagnosis to surgery, visual analog scale (VAS) pain score, Cooper Scale grade, modified Japanese Orthopedic Association score (mJOA), white blood cell count, and C-reactive protein (CRP). Assessments were performed preoperatively (T0), 1 week after surgery (T1), and at 6 months (T2). Imaging findings from representative cases were reviewed to illustrate the radiographic evolution after decompression combined with antibiotic therapy and orthotic support. **Results**: The cohort included 8 women and 7 men with a mean age of 57.33 years. Twelve patients (80%) underwent surgery within 48 h because of neurological deterioration or urgent compressive findings. Thoracic involvement was predominant (11 cases, 73%), whereas cervical and lumbar disease accounted for 1 and 3 cases, respectively. Intraoperative cultures were positive in 9 patients (60%), and *Staphylococcus aureus* was isolated in 7 of these 9 cases (77.8%). Mean VAS pain score improved from 6.3 at T0 to 4.3 at T1 and 1.4 at T2. Mean lower-extremity Cooper Scale grade improved from 2.7 to 2.1 to 0.6; mean upper-extremity Cooper Scale grade improved from 1.3 to 1.0 to 0. Mean mJOA improved from 10.3 to 11.4 to 16.0. Mean white blood cell count declined from 11.58 to 8.35 to 6.14 × 10^3^/µL, and mean CRP declined from 123.9 to 72.81 to 19.44 mg/L. No patient worsened neurologically after surgery. No patient required acute stabilization or delayed arthrodesis during follow-up. **Conclusions**: In our case series of patients with compressive or progressive neurological spondylodiscitis, decompressive laminectomy combined with antibiotic treatment, bracing, and rehabilitation was associated with early neurological stabilization, substantial pain relief, and marked reduction in inflammatory markers, without subsequent need for instrumented stabilization. These data support the role of spinal decompression as part of a personalized, effective treatment strategy in patients with neurological decline.

## 1. Introduction

Spondylodiscitis remains one of the most challenging infectious conditions encountered in spinal surgery because diagnosis is often delayed, clinical presentation is highly heterogeneous, and surgical indications vary substantially among patients. Consequently, management should be individualized according to the patient’s clinical presentation, neurological status, radiological findings, and overall medical condition.

The incidence of native vertebral osteomyelitis has increased over recent decades, particularly among older adults and patients with multiple comorbidities, immunosuppression, previous invasive spinal procedures, or bloodstream infections. Although hematogenous dissemination remains the predominant route of infection, postoperative and other iatrogenic forms have become increasingly prevalent, paralleling the growing number of spinal interventions performed worldwide [1,2,3].

*Staphylococcus aureus* is the most frequently isolated pathogen in pyogenic spondylodiscitis, with methicillin-susceptible strains accounting for most cases, although methicillin-resistant strains are being identified with increasing frequency. Other commonly implicated organisms include *Streptococcus* spp., coagulase-negative staphylococci, members of the Enterobacteriaceae family—particularly *Escherichia coli*—*Pseudomonas aeruginosa*, and *Enterococcus* spp.

Tuberculous spondylodiscitis occurs almost exclusively through hematogenous dissemination and is observed predominantly in immunocompromised individuals and patients originating from regions where tuberculosis is endemic, including sub-Saharan Africa and the Indian subcontinent. A previous history of tuberculosis or concomitant active infection, most commonly involving the pulmonary or genitourinary systems, is reported in approximately one-third to one-half of affected patients [4].

The pathophysiology of spondylodiscitis underlies its potentially severe clinical course. Infection involving the vertebral endplates and intervertebral disc may progress from bone marrow edema and disc destruction to paravertebral phlegmon, epidural abscess formation, vertebral body collapse, segmental deformity, and neural compression [5,6,7,8]. Pain is the most consistent presenting symptom, whereas fever is inconsistently observed and laboratory abnormalities are often nonspecific. Leukocytosis may be absent even in advanced disease, while C-reactive protein (CRP) is generally more sensitive for both diagnosis and treatment monitoring [7,9,10]. Although procalcitonin (PCT) appears to have a more limited diagnostic value than CRP and erythrocyte sedimentation rate (ESR), serum levels exceeding 0.11 ng/mL have been associated with poorer clinical outcomes, suggesting a predominantly prognostic rather than diagnostic role. Owing to this frequently indolent presentation, diagnosis is often delayed for several weeks, increasing the risk of irreversible neurological deficits and long-term disability [11,12].

Magnetic resonance imaging (MRI) is the imaging modality of choice because it enables early detection of marrow and disc signal abnormalities while accurately delineating epidural and paravertebral extension, with excellent sensitivity and specificity [7,9,13]. Computed tomography (CT) provides complementary information by demonstrating endplate erosion and osseous destruction and by facilitating image-guided percutaneous biopsy. In selected cases, fluorodeoxyglucose positron emission tomography/computed tomography (FDG-PET/CT) may be particularly valuable when MRI findings are inconclusive or diagnostic uncertainty persists [7,13,14,15]. The management of patients presenting with rapidly progressive myelopathy or cauda equina syndrome represents a particularly challenging scenario, as the urgency of surgical decompression must be carefully balanced against the need to establish a definitive microbiological diagnosis whenever feasible [4,11].

Current evidence emphasizes the importance of obtaining microbiological confirmation whenever possible and administering prolonged pathogen-directed antimicrobial therapy [7,9,16]. Nevertheless, surgical intervention remains essential in selected patients, particularly those presenting with epidural abscess, progressive neurological deterioration, spinal instability, progressive deformity, or failure of conservative treatment [4,6,7,8,17,18,19,20]. The optimal surgical strategy, however, remains a matter of debate. Most published studies have focused on debridement combined with instrumented stabilization or anterior–posterior reconstruction, especially in patients with unstable thoracolumbar disease [4,8,18,20,21,22]. In contrast, relatively few investigations have specifically evaluated decompressive laminectomy as the primary surgical approach in patients whose principal indication for surgery is neural compression rather than mechanical instability [4,6,7,8,9,13,15,17,18,21,22,23]. Accordingly, decompressive laminectomy without concomitant stabilization should be reserved for carefully selected patients requiring posterior neural decompression and epidural abscess drainage in the absence of significant spinal instability or deformity [9,24].

The present single-center retrospective study therefore evaluated a selected cohort of patients with spondylodiscitis treated with decompressive laminectomy without immediate instrumented stabilization. The objective was neither to compare surgical and conservative management nor to suggest equivalence with reconstructive procedures in patients with unstable disease. Rather, the study sought to evaluate the clinical outcomes of spinal decompression in patients presenting with spinal canal compromise and neurological deficits. We hypothesized that, in carefully selected patients, decompressive laminectomy combined with targeted antimicrobial therapy, external spinal bracing, and structured rehabilitation would result in meaningful improvements in pain, neurological function, functional independence, and inflammatory markers while avoiding the need for subsequent instrumented arthrodesis.

## 2. Materials and Methods

This retrospective single-center case series was conducted at the University Hospital of Trieste. Electronic inpatient and outpatient medical records were systematically reviewed to identify all consecutive patients with a clinical, laboratory, and radiological diagnosis of spondylodiscitis who underwent decompressive laminectomy between January 2017 and December 2023. During the study period, 98 patients were diagnosed with spondylodiscitis. Of these, four patients (4.1%) underwent instrumented spinal stabilization (three lumbar and one cervical) and were excluded from the analysis. Fifteen patients (15.3%) underwent decompressive surgery without concomitant stabilization and constituted the study cohort. Patients were eligible if they met the following criteria: (1) confirmed diagnosis of spondylodiscitis; (2) neurological deterioration attributable to the infection, manifesting as myelopathy and/or radiculopathy; (3) surgical decompression of the spinal canal by laminectomy or hemilaminectomy; and (4) a minimum clinical and laboratory follow-up of six months. Exclusion criteria included (1) acute instrumented spinal stabilization and (2) follow-up shorter than six months. Ethical review and approval were waived for this study by the Committee of the Cattinara Hospital of Trieste due to the retrospective nature of the study. Informed consent for research and publication of anonymized data was obtained from all patients involved in this study.

Surgical decision-making reflected routine neurosurgical practice rather than a predefined treatment algorithm. Decompressive laminectomy was indicated in patients presenting with neurological compromise secondary to spondylodiscitis, including myelopathy and/or radiculopathy caused primarily by epidural or posterior extension of the infectious process. Neural compression most commonly resulted from epidural abscess formation or inflammatory tissue extending from the infected intervertebral disc. Instrumented stabilization was reserved for patients demonstrating radiological evidence of mechanical instability on preoperative computed tomography (CT) and MRI. Criteria for stabilization included progressive vertebral body collapse, significant segmental kyphotic deformity, pathological vertebral translation or subluxation, and extensive destruction of the anterior spinal column. In the absence of these findings, decompression alone was considered an appropriate surgical strategy when neural compression represented the principal indication for intervention.

MRI was performed in all patients and served as the primary imaging modality for surgical planning. Intraoperative specimens were routinely obtained for microbiological culture before initiation or modification of antimicrobial therapy whenever feasible.

For each patient, demographic, clinical, laboratory, and radiological data were retrospectively collected. Variables of interest included the spinal level involved, interval between diagnosis and surgery, pain intensity, neurological status, functional outcome, white blood cell (WBC) count, and CRP level.

Time from diagnosis to surgery was recorded in hours or days and subsequently categorized as urgent (<48 h) or delayed (≥48 h). Clinical assessments were performed at three predefined time points: preoperatively (T0), one week after surgery (T1), and six months postoperatively (T2). Pain intensity was assessed using the VAS. Neurological impairment was graded using the Cooper Scale, which evaluates upper- and lower-extremity motor function on a five-point ordinal scale, with lower scores indicating better neurological function (1 = no impairment; 5 = complete paralysis). Because the study population included cervical, thoracic, and lumbar infections, upper- and lower-extremity Cooper scores were analyzed separately. Functional neurological status was evaluated using the modified Japanese Orthopaedic Association (mJOA) score, which assesses upper- and lower-extremity motor function, upper-extremity sensory function, and bladder function. Total scores range from 0 to 18, with higher values indicating better neurological performance. Both the Cooper Scale and mJOA scores were retrospectively reconstructed from standardized neurological examinations documented in the medical records. Laboratory parameters were extracted directly from the institutional electronic database. Representative imaging studies were reviewed qualitatively to illustrate the extent of preoperative spinal canal compromise and postoperative radiological evolution following decompression and antimicrobial treatment.

Given the limited sample size and the exploratory nature of this study, statistical analyses were primarily descriptive. Continuous variables are reported as mean values, whereas categorical variables are presented as absolute frequencies and percentages. No formal inferential statistical analyses were performed because of the small cohort and the absence of an appropriate comparison group. Accordingly, *p*-values and measures of statistical significance were not calculated. The findings should therefore be interpreted as those of a descriptive surgical case series intended to characterize clinical outcomes and inform surgical decision-making rather than establish causal relationships or comparative treatment efficacy.

## 3. Results

Fifteen patients met the predefined inclusion criteria and constituted the study cohort (Table 1). The cohort included eight women (53.3%) and seven men (46.7%), with a mean age of 57.3 ± 17.7 years.

Surgical decompression was performed on an urgent basis in most patients. Twelve patients (80%) underwent surgery within 48 h of neurological deterioration. Although the interval between the initial diagnosis of spondylodiscitis and the onset of neurological symptoms could not be reliably determined because of the retrospective study design, the time from recognition of neurological deterioration to surgical intervention was available for all patients. Among those undergoing urgent surgery, the mean interval between symptom onset and decompression was 15.3 h. The remaining three patients (20%) underwent delayed surgery after 5, 7, and 14 days, respectively. In these cases, neurological deficits had already been present for more than one week at the time of neurosurgical consultation, and initial management prioritized stabilization of systemic septic complications before surgical treatment.

The surgical approach consisted of bilateral laminectomy in eight patients (53.3%) and hemilaminectomy in seven (46.7%). The extent of decompression was individualized according to the location and distribution of neural compression. Hemilaminectomy was preferentially performed in patients with unilateral canal compromise to minimize surgical invasiveness. Bilateral decompression involved a maximum of three contiguous vertebral levels, whereas unilateral decompression was performed across as many as ten consecutive levels (T9–S1) in cases of extensive posterior epidural involvement.

Preoperative antimicrobial management was tailored to the patient’s clinical condition, microbiological findings, and severity of systemic infection. When clinically feasible, initiation of empirical antibiotic therapy was deferred until intraoperative microbiological specimens had been obtained in order to maximize culture yield. However, patients presenting with severe sepsis or systemic instability received immediate empirical intravenous antimicrobial therapy before surgery.

The thoracic spine was the most frequently affected anatomical region, being involved in 11 of the 15 patients (73.3%). Infection was confined to the thoracic spine in six patients, extended to the cervicothoracic junction in three, and involved the thoracolumbar region in two. Isolated lumbar spondylodiscitis was observed in three patients (20.0%), whereas only one patient (6.7%) presented with isolated cervical disease.

Microbiological confirmation was obtained in nine patients (60.0%). *Staphylococcus aureus* was identified in seven cases (77.8%), confirming its predominant etiological role in pyogenic spondylodiscitis. The remaining positive cultures yielded Klebsiella pneumoniae and *Streptococcus* intermedius, each isolated in one patient.

Antimicrobial therapy was tailored according to microbiological culture and antimicrobial susceptibility testing whenever a causative pathogen was identified. In patients requiring empirical treatment, broad-spectrum intravenous antibiotics providing coverage against methicillin-resistant *Staphylococcus aureus* and Gram-negative organisms were initiated and subsequently de-escalated once microbiological results became available. Whenever clinically feasible, intravenous therapy was transitioned early to an appropriate oral regimen. The overall duration of antimicrobial treatment was individualized according to clinical response, laboratory markers, and adequacy of surgical source control; however, treatment generally continued for a minimum of eight weeks.

Postoperative rehabilitation differed from conventional protocols following elective spinal surgery, in which early mobilization is typically encouraged. Given the underlying infectious pathology and the need to minimize mechanical stress on the affected spinal segment, patients were managed with an extended period of bed rest followed by gradual mobilization using external spinal orthoses. Brace selection was individualized according to the anatomical location of the infection and the extent of surgical decompression. Rigid thoracic orthoses were prescribed for thoracic lesions, semirigid lumbar braces with posterior support for lumbar involvement, and semirigid cervical collars for cervical disease. External immobilization was maintained for at least three months in all patients.

Postoperative MRI examinations obtained one week and six months after surgery were systematically reviewed to evaluate the evolution of the infectious process and to detect radiographic evidence of delayed spinal instability. Throughout follow-up, none of the patients demonstrated imaging findings suggestive of progressive mechanical instability, including vertebral body collapse, increasing segmental kyphotic deformity, pathological vertebral translation, spondylolisthesis, or progressive sagittal imbalance.

Pain relief was one of the most consistent clinical outcomes observed following decompressive surgery (Table 2).

Mean VAS scores decreased from 6.3 preoperatively to 4.3 one week after surgery and further declined to 1.4 at the six-month follow-up. No patient experienced worsening pain during the early postoperative period, and all patients reported lower pain scores at six months than at baseline.

At the final follow-up, complete pain resolution (VAS score = 0) was achieved in seven patients (46.7%). Six patients (40.0%) reported only mild residual pain (VAS score 1–3), whereas moderate residual pain (VAS score 4–7) persisted in two patients (13.3%), both of whom nonetheless demonstrated substantial improvement compared with their preoperative condition. Early clinical benefit was already evident one week after surgery in 13 patients (86.7%), while pain scores remained unchanged in the remaining two patients.

**Figure 1:**
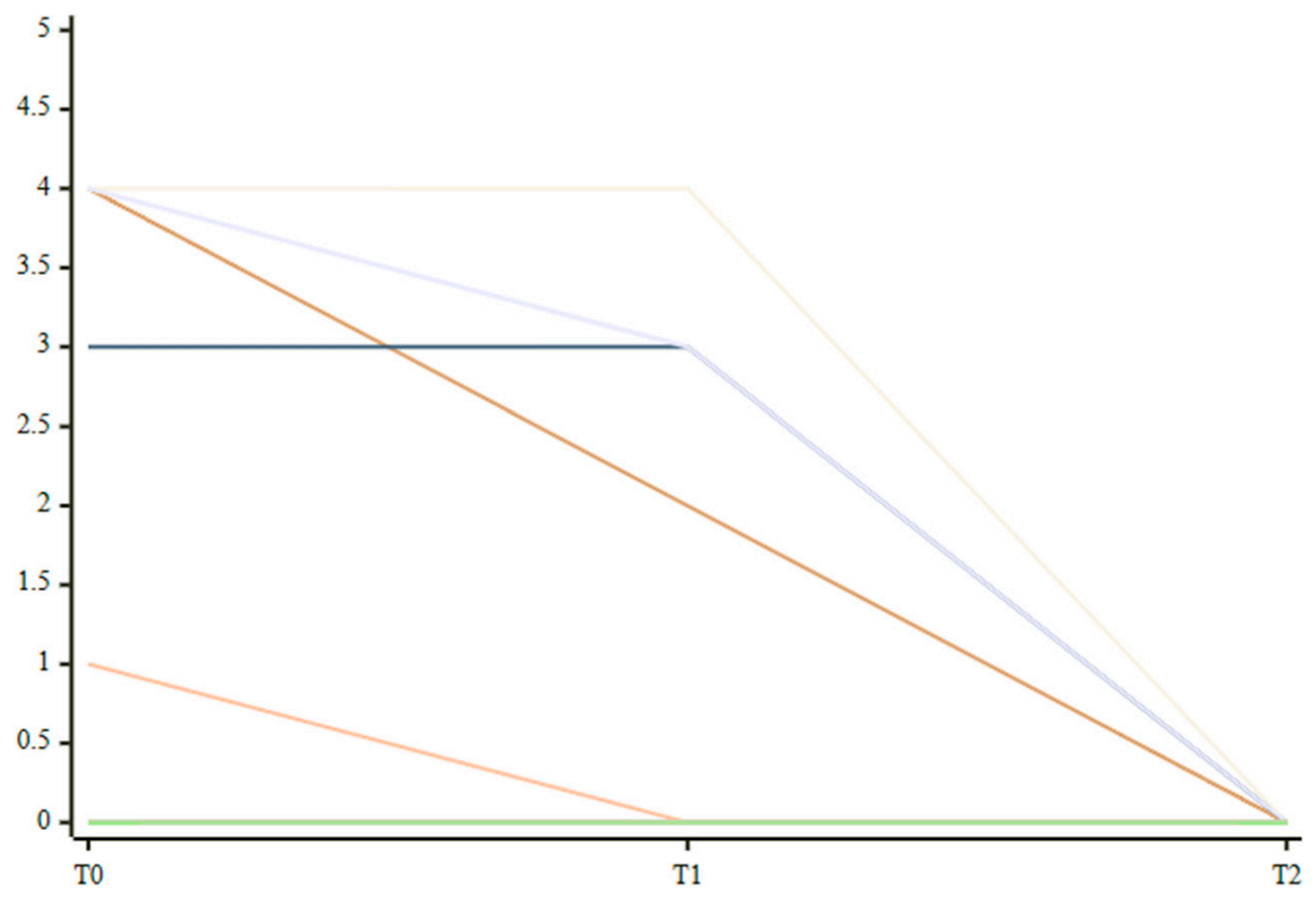
Cooper Scale trend at individual level, at T0–T1–T2 for upper limbs.

**Figure 2:**
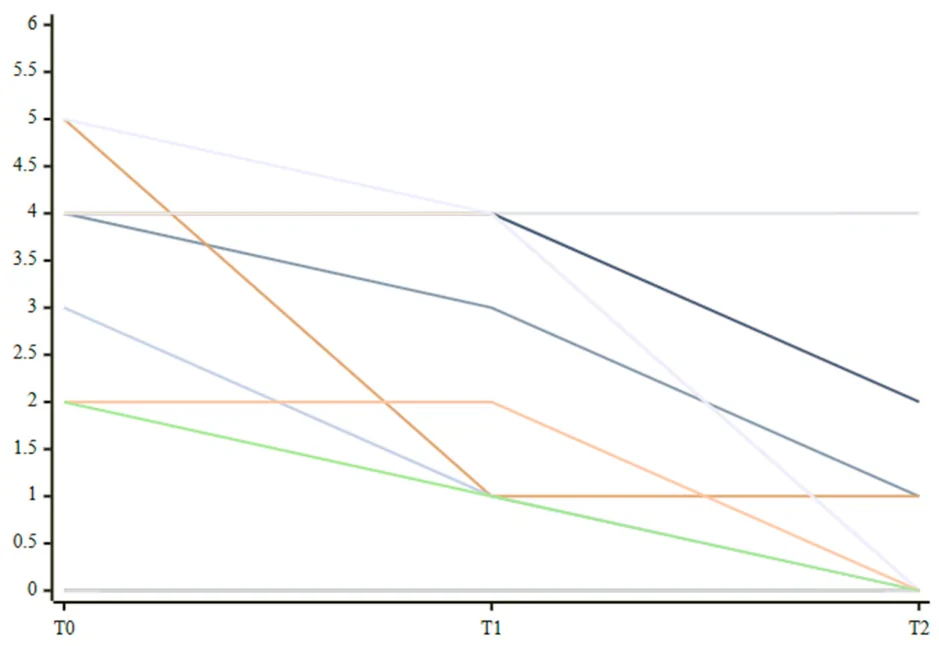
Cooper Scale trend at individual level, at T0–T1–T2 for lower limbs.

Neurological recovery paralleled the improvement in pain. Mean lower-extremity Cooper Scale scores decreased from 2.7 at baseline to 2.1 at one week and 0.6 at six months (Figure 1). Similarly, mean upper-extremity Cooper Scale scores improved from 1.3 preoperatively to 1.0 at one week and reached 0.0 at the final follow-up (Figure 2). Notably, no patient experienced postoperative neurological deterioration.

Overall, neurological function improved in 11 patients (73.3%) during follow-up, whereas four patients (26.7%) maintained stable neurological status throughout the study period. Among patients demonstrating neurological recovery, six (54.5%) exhibited measurable improvement as early as the first postoperative week, while the remaining patients showed progressive recovery over the subsequent months.

**Figure 3:**
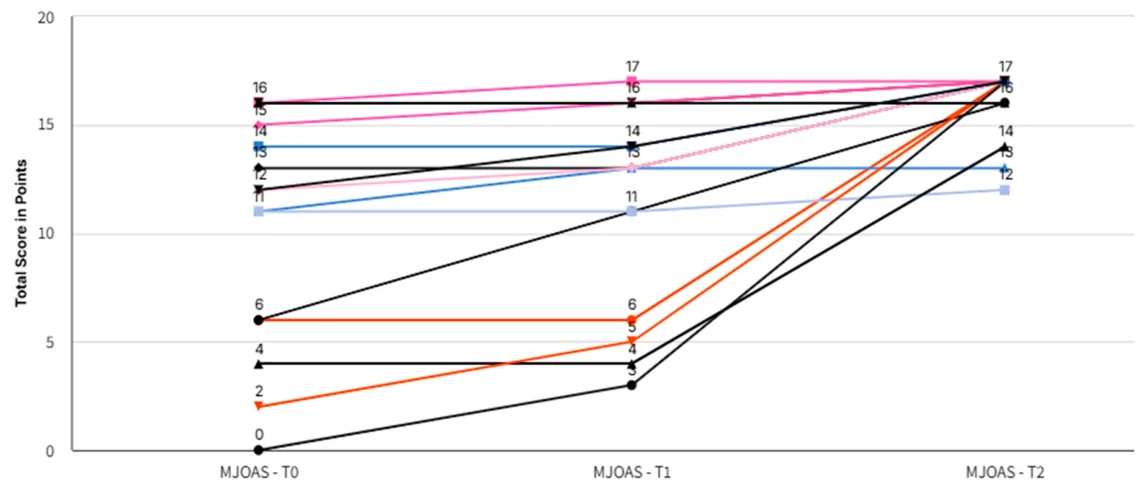
Modified Japanese Orthopedic Association Score (mJOAS) graphical trend at individual level, at T0–T1–T2.

Functional outcomes, as assessed by the modified Japanese Orthopaedic Association (mJOA) score, paralleled the improvements observed in pain and neurological status (Figure 3). The mean mJOA score increased from 10.3 preoperatively to 11.4 at one week and reached 16.0 at the six-month follow-up. All patients demonstrated functional improvement by the final evaluation, while eight patients (53.3%) had already shown measurable gains during the first postoperative week.

Patients presenting with more severe neurological impairment, including those with combined upper- and lower-extremity deficits, exhibited marked functional recovery over the course of follow-up. No patient experienced deterioration in mJOA score at any postoperative assessment.

Inflammatory markers showed a progressive decline throughout follow-up. Mean WBC count decreased from 11.58 × 10^3^/µL preoperatively to 8.35 × 10^3^/µL one week after surgery and 6.14 × 10^3^/µL at the six-month follow-up. Baseline leukocytosis (>11.0 × 10^3^/µL) was present in only five patients (33.3%), whereas two patients (13.3%) had leukopenia and eight (53.3%) exhibited WBC counts within the normal reference range despite active infection.

In contrast, CRP was elevated in most patients at presentation and demonstrated a more consistent response to treatment. Mean CRP levels declined from 123.9 mg/L at baseline to 72.8 mg/L one week after surgery and 19.4 mg/L at six months. CRP values decreased during the first postoperative week in 12 patients (80.0%), and all patients exhibited lower CRP levels at the final follow-up than at baseline.

**Figure 4:**
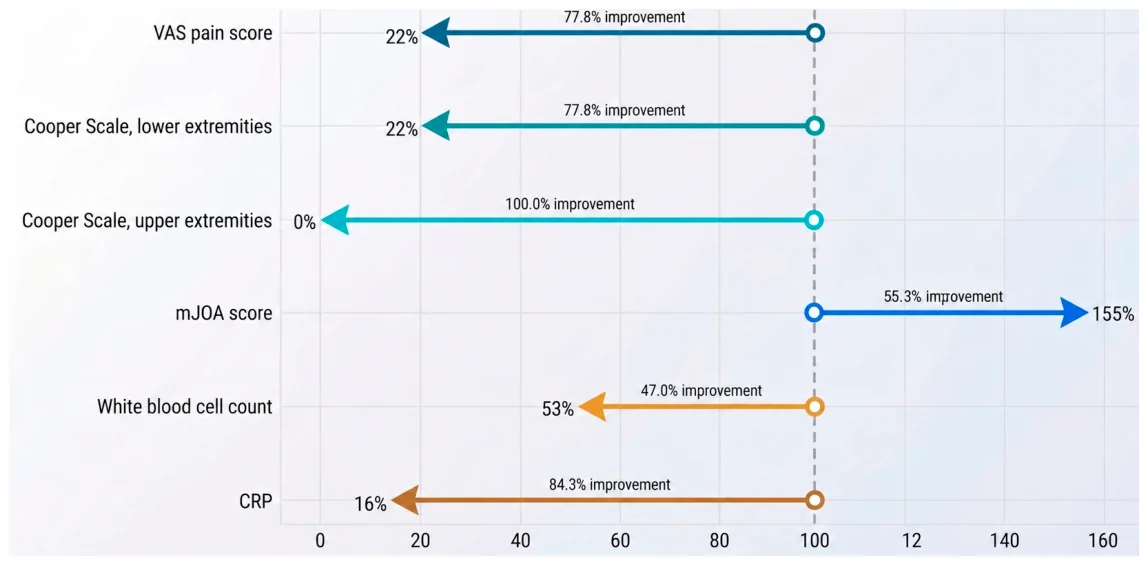
Percentage change in clinical, neurological, and laboratory outcomes from preoperative baseline to 6-month follow-up. For VAS, the Cooper scale, white blood cell count, and CRP, improvement is indicated by a reduction from baseline values, whereas for mJOA, it is indicated by an increase from baseline. Abbreviations: CRP, C-reactive protein; mJOA, Modified Japanese Orthopedic Association; VAS, visual analog score.

Overall, sustained clinical and laboratory improvement was observed during the six-month follow-up period (Figure 4). Compared with baseline, mean VAS scores decreased by 77.8%, lower-extremity Cooper Scale scores by 77.8%, and upper-extremity Cooper Scale scores by 100.0%. Mean mJOA scores increased by 55.3%, reflecting substantial functional neurological recovery. Laboratory parameters demonstrated a similar trend, with mean WBC counts decreasing by 47.0% and mean CRP levels by 84.3% relative to preoperative values.

**Figure 5:**
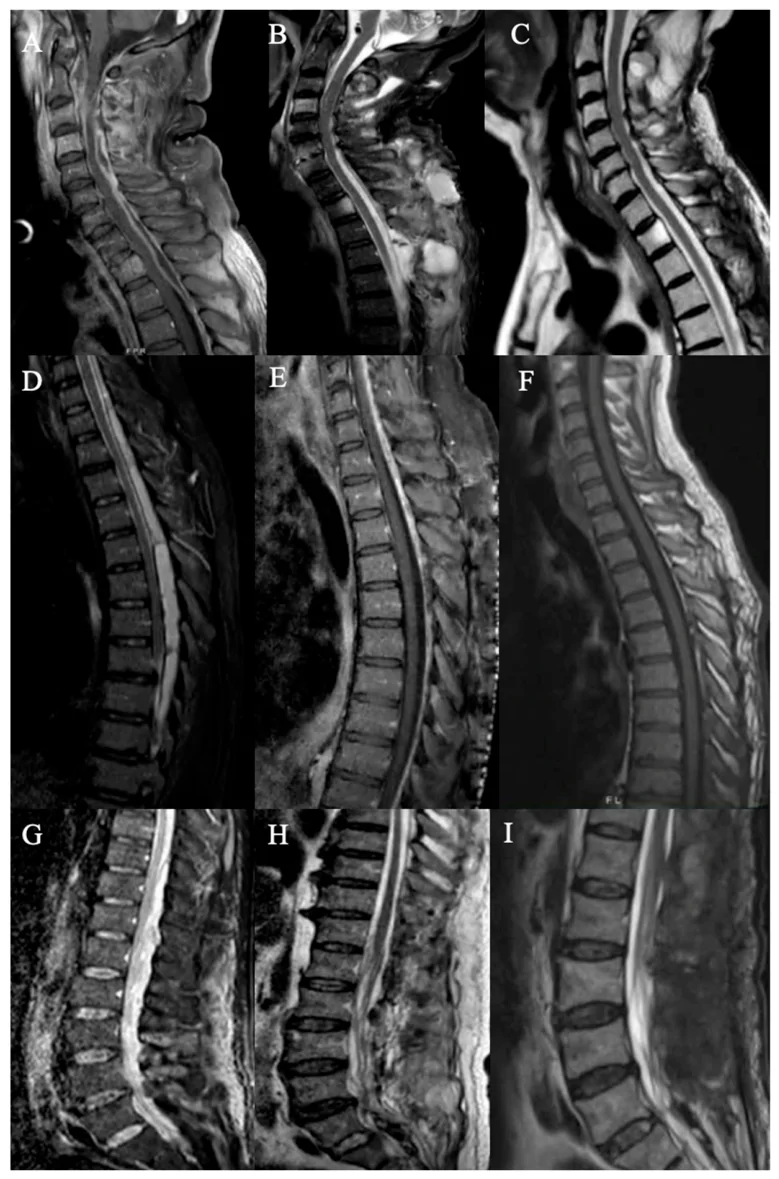
Preoperative, early postoperative, and 6-month postoperative sagittal MRI scan of 3 representative cases of spinal epidural abscess due to spondylodiscitis, successfully treated with emi-laminectomy. The first case (panels (**A**–**C**)) is a 68-year-old male with cervical (C4–C6) and thoracic (T3–T5) epidural abscess. The second case (panels (**D**–**F**)) is a 39-year-old male with thoracic (T5–T10) epidural abscess. The third case (panels (**G**–**I**)) is a 73-year-old male with thoracolumbar (T9–S1) epidural abscess. Across all 3 cases, early postoperative MRI demonstrated restoration of canal patency, and 6-month imaging showed maintenance of decompression with resolution of the compressive component despite residual structural changes typical of healed infection.

As defined by the study protocol, no patient underwent instrumented stabilization during the index procedure. Furthermore, no patient required delayed spinal fusion or secondary arthrodesis during the six-month follow-up, despite several patients having undergone multilevel laminectomy or hemilaminectomy (Figure 5). Serial postoperative imaging revealed no evidence of progressive mechanical instability requiring secondary instrumentation.

## 4. Discussion

The findings of the present study suggest that the timing of surgical intervention should be guided by both the severity of neurological impairment and the rate of clinical progression. Patients presenting with rapidly progressive motor deficits or severe neurological compromise are likely to benefit from timely surgical decompression, provided that the neurological deficit has not been established for more than 7–10 days, beyond which functional recovery is generally considered less likely [9].

Pain outcomes were consistently favorable in the present cohort. No patient experienced worsening pain during the first postoperative week, and all patients demonstrated sustained improvement at the six-month follow-up. Although the limited sample size precludes definitive conclusions, these findings support the role of early decompressive surgery in providing rapid pain relief while preventing further neurological deterioration in carefully selected patients.

Neurological and functional outcomes showed a similarly favorable pattern. Functional status, as assessed by the mJOA score, improved in all patients by the six-month follow-up, with more than half demonstrating measurable recovery within the first postoperative week. Likewise, Cooper Scale assessments showed no postoperative neurological deterioration, and neurological function improved in 11 of the 15 patients during follow-up. Among these patients, more than half exhibited early neurological recovery within the first postoperative week, whereas the remainder showed progressive improvement over the subsequent months.

Collectively, these findings suggest that timely decompressive surgery, when performed in appropriately selected patients, may promote meaningful neurological recovery and improved functional independence in addition to effective pain control.

Evidence specifically addressing decompression without concomitant spinal stabilization in spondylodiscitis remains limited. Karadimas et al. [25] reported favorable neurological recovery in five of eleven surgically treated patients, whereas Noh et al. [26] identified decompression alone as an effective strategy for pain relief. Current recommendations support urgent surgical evaluation and decompression in patients with spinal cord or nerve root compression associated with progressive neurological deficits; however, the benefit of surgery for long-standing established deficits remains uncertain, particularly when neurological impairment has been present for more than 48 h [27].

The anatomical distribution of infection in the present cohort differs from that reported in large epidemiological studies, in which lumbar spondylodiscitis predominates [5,6,7,15,21]. This discrepancy is likely explained by the study’s surgical selection criteria rather than by differences in disease epidemiology. Thoracic epidural extension is more likely to cause spinal cord compression requiring urgent decompression, whereas lumbar disease more commonly presents with pain or isolated radiculopathy and is often managed conservatively. In addition, the inherent biomechanical stability and rigidity of the thoracic spine may reduce the need for instrumented stabilization compared with the cervical and lumbar regions [4,5,6,11].

Microbiological confirmation was achieved in nine patients (60%) through intraoperative tissue sampling, while cultures remained negative in the remaining six patients (40%). This finding is consistent with previous reports highlighting the limited diagnostic yield of both image-guided percutaneous biopsy and surgical sampling in vertebral osteomyelitis [5,7,9,15,28]. False-negative cultures may result from prior antimicrobial therapy, low bacterial burden, sampling error, or preanalytical factors affecting specimen quality and processing. The diagnostic yield of percutaneous biopsy also depends on the anatomical site sampled, with discal, epidural, and paravertebral specimens generally providing higher culture positivity than vertebral body samples alone. Although surgical sampling offers the advantage of direct visualization and permits collection of larger and multiple tissue specimens, it does not guarantee microbiological confirmation and is limited to patients with a surgical indication or unsuccessful percutaneous biopsy [4,5,9,11,16,29].

All patients were managed with external spinal bracing throughout the postoperative period and for at least three months thereafter. Orthotic treatment facilitates protected mobilization, may reduce pain, and limits mechanical stress on the infected spinal segment while biological healing occurs [30]. Notably, no patient required delayed spinal stabilization during follow-up despite several undergoing multilevel decompression. These findings are consistent with previous reports suggesting that, in appropriately selected patients without preoperative mechanical instability, infection resolution combined with external immobilization may allow sufficient structural remodeling to restore spinal stability [31]. Consequently, instrumented stabilization should remain reserved for patients with established or progressive mechanical instability, vertebral collapse, significant deformity, sagittal imbalance, or persistent mechanical pain after eradication of the infection.

Laboratory findings further supported the clinical evolution observed in this cohort. At presentation, CRP levels were elevated in most patients, whereas leukocytosis was absent in a substantial proportion, consistent with previous studies demonstrating the limited sensitivity of WBC count in vertebral osteomyelitis [7,9,10,32]. Although interpretation of inflammatory markers during the early postoperative period should consider the physiological inflammatory response to surgery, both CRP and WBC values showed a progressive decline throughout follow-up. By six months, CRP levels had decreased in all patients, while WBC counts improved in 13 of the 15 patients.

Taken together, these findings reinforce the role of CRP as the most useful laboratory marker for both the diagnosis and longitudinal monitoring of spondylodiscitis. Unlike WBC count, which may remain within normal limits despite active infection, serial CRP measurements closely paralleled clinical recovery and therefore appear particularly valuable for assessing treatment response during both the postoperative period and long-term follow-up [7,21,22,33].

## 5. Limitations

Several limitations should be considered when interpreting the findings of the present study. First, the retrospective single-center design and the relatively small sample size limit the statistical power and external validity of the results. However, the limited number of patients reflects the highly selective inclusion criteria, which were designed to evaluate a homogeneous cohort of patients with spondylodiscitis undergoing decompressive laminectomy without concomitant spinal stabilization.

The inclusion of patients with cervical, thoracic, and lumbar disease introduced clinical heterogeneity that may have influenced outcomes. Nevertheless, this heterogeneity also reflects routine neurosurgical practice and provides preliminary evidence that decompressive surgery may be applicable across different spinal regions when appropriately indicated. As with all retrospective surgical series, some degree of selection bias was unavoidable. Patients deemed unsuitable for surgery because of severe comorbidities, those who declined operative treatment, and those in whom surgery could not be performed despite an appropriate indication were not represented in the study cohort.

Another limitation is the absence of inferential statistical analyses. Owing to the exploratory nature of the study and the limited sample size, only descriptive statistics were reported, precluding formal comparisons and limiting the strength of the conclusions. In addition, although a six-month follow-up was sufficient to assess early neurological recovery, pain relief, and normalization of inflammatory markers, it does not permit definitive evaluation of long-term spinal stability or late mechanical complications.

Prospective multicenter studies involving larger patient populations and longer clinical and radiological follow-up are needed to validate these findings, determine whether outcomes differ according to the spinal level involved, and establish the long-term durability of decompression without instrumented stabilization. Follow-up extending to at least 12–24 months would provide a more robust assessment of spinal stability and the potential need for delayed fusion.

## 6. Conclusions

The findings of the present study suggest that decompressive laminectomy represents a valuable treatment option for carefully selected patients with spondylodiscitis who develop progressive neurological impairment requiring timely surgical intervention. When combined with appropriate antimicrobial therapy, external spinal bracing, and structured rehabilitation, decompression alone was associated with favorable clinical outcomes without the need for immediate or delayed instrumented stabilization in this cohort.

Spinal stabilization should not be considered mandatory in all surgically treated patients with spondylodiscitis but rather reserved for those with preexisting or progressive mechanical instability, significant deformity, or extensive vertebral destruction. Consequently, surgical management should be individualized according to neurological status, radiological evidence of instability, extent of infection, patient comorbidities, and overall clinical condition. In this single-center series, decompressive surgery was associated with sustained improvements in pain, neurological function, functional recovery, and inflammatory markers, with no cases of postoperative neurological deterioration or delayed spinal fusion during follow-up.

Prospective multicenter comparative studies with larger patient populations are warranted to identify the patients most likely to benefit from decompression alone and to define the optimal extent of decompression, as well as the indications for immediate reconstruction with or without instrumented stabilization.

## Figures and Tables

**Table 1:** Baseline characteristics of the decompressive laminectomy cohort.

Variable	Value
Patients	15
Sex	8 female (53%), 7 male (47%)
Age	Mean 57.33 ± 17.66 years
Urgent surgery (<48 h)	12 (80%)
Delayed surgery (>48 h)	3 (20%)
Anatomical distribution	Cervical 1 (6.7%), thoracic 11 (73.3%), lumbar 3 (20.0%)
Thoracic extension pattern	6 thoracic only; 3 cervicothoracic; 2 thoracolumbar
Microbiological diagnosis	Positive intraoperative cultures in 9 (60%)
Identified Pathogens	*Staphylococcus aureus* in 7/9 culture-positive cases (77.8%) *Streptococcus Intermedius* in 1/8 culture-positive cases (12.5%) *Klebsiella Pneumoniae* in 1/8 culture-positive cases (12.5%)
Index stabilization	None
Delayed arthrodesis during follow-up	None

**Table 2:** Longitudinal clinical and laboratory outcomes.

Outcome	T0 (Preop)	T1 (1 Week)	T2 (6 Months)
Mean VAS	6.3	4.3	1.4
Mean Cooper Scale, lower extremities	2.7	2.1	0.6
Mean Cooper Scale, upper extremities	1.3	1.0	0.0
Mean mJOA score	10.3	11.4	16.0
Mean white blood cell count (×10^3^/µL)	11.58	8.35	6.14
Mean CRP (mg/L)	123.9	72.81	19.44

Abbreviations: CRP, C-reactive protein; mJOA, Modified Japanese Orthopedic Association; VAS, visual analog score.

## Data Availability

The raw data supporting the conclusions of this article will be made available by the authors on request.

## Abbreviations

CRP	C-reactive protein
CT	Computer tomography
CS	Cooper Scale
FDG-PET/CT	Fluorodeoxyglucose positron emission tomography/computed tomography
MRI	Magnetic resonance imaging
mJOA	Modified Japanese Orthopedic Association
mJOAS	Modified Japanese Orthopedic Association score
VAS	Visual analog scale pain score
